# Supplementary material for: 1 °C warming increases spatial competition frequency and complexity in Antarctic marine macrofauna
Source: Commun Biol. 2021 Feb 16;4:208. doi: 10.1038/s42003-021-01742-w (PMC7886862; doi:10.1038/s42003-021-01742-w)
Supplement: Supplementary file 2 — Reporting Summary [file 42003_2021_1742_MOESM2_ESM.pdf]

nature research

Corresponding author(s): D K A Barnes

Last updated by author(s): Dec 17, 2020

Reporting Summary

Nature Research wishes to improve the reproducibility of the work that we publish. This form provides structure for consistency and transparency in reporting. For further information on Nature Research policies, see our Editorial Policies and the Editorial Policy Checklist.

Statistics

For all statistical analyses, confirm that the following items are present in the figure legend, table legend, main text, or Methods section.

n/a	Confirmed	
☐	☒	The exact sample size (n) for each experimental group/condition, given as a discrete number and unit of measurement
☒	☐	A statement on whether measurements were taken from distinct samples or whether the same sample was measured repeatedly
☐	☒	The statistical test(s) used AND whether they are one- or two-sided<br>*Only common tests should be described solely by name; describe more complex techniques in the Methods section.*
☒	☐	A description of all covariates tested
☐	☒	A description of any assumptions or corrections, such as tests of normality and adjustment for multiple comparisons
☐	☒	A full description of the statistical parameters including central tendency (e.g. means) or other basic estimates (e.g. regression coefficient) AND variation (e.g. standard deviation) or associated estimates of uncertainty (e.g. confidence intervals)
☐	☒	For null hypothesis testing, the test statistic (e.g. F, t, r) with confidence intervals, effect sizes, degrees of freedom and P value noted<br>*Give P values as exact values whenever suitable.*
☒	☐	For Bayesian analysis, information on the choice of priors and Markov chain Monte Carlo settings
☒	☐	For hierarchical and complex designs, identification of the appropriate level for tests and full reporting of outcomes
☒	☐	Estimates of effect sizes (e.g. Cohen's d, Pearson's r), indicating how they were calculated

Our web collection on statistics for biologists contains articles on many of the points above.

Software and code

Policy information about availability of computer code

Data collection	No software used for the collection of this data
Data analysis	Staistical tests were completed using R software version 3.6.1, specifically the userfriendlyscience package version 0.7.2 and Vegan package version 2.5-6. Pairwise community composition tests and SIMPER analysis were completed in PRIMER 6.

For manuscripts utilizing custom algorithms or software that are central to the research but not yet described in published literature, software must be made available to editors and reviewers. We strongly encourage code deposition in a community repository (e.g. GitHub). See the Nature Research guidelines for submitting code & software for further information.

Data

Policy information about availability of data

All manuscripts must include a data availability statement. This statement should provide the following information, where applicable:

- Accession codes, unique identifiers, or web links for publicly available datasets
- A list of figures that have associated raw data
- A description of any restrictions on data availability

The original raw data are available online at https://github.com/Gaton1/Communications-Biology-Barnes-2020

Field-specific reporting

Please select the one below that is the best fit for your research. If you are not sure, read the appropriate sections before making your selection.

☒ Life sciences ☐ Behavioural & social sciences ☐ Ecological, evolutionary & environmental sciences

For a reference copy of the document with all sections, see nature.com/documents/nr-reporting-summary-flat.pdf

Life sciences study design

All studies must disclose on these points even when the disclosure is negative.

Sample size	Sample size was chosen based in prior working experience with panels at this location and the practical limitations of working underwater in polar locations. The presence of statistically significant results confirms adequate sample size.
Data exclusions	No date were excluded from the analysis
Replication	The reproducibility of the results was confirmed by using similar panels at the same location at a prior time
Randomization	1 panel per treatment was randomly positioned (using a random number generator) for each treatment block
Blinding	Investigators were blinded to group allocation during analysis

Reporting for specific materials, systems and methods

We require information from authors about some types of materials, experimental systems and methods used in many studies. Here, indicate whether each material, system or method listed is relevant to your study. If you are not sure if a list item applies to your research, read the appropriate section before selecting a response.

Materials & experimental systems

n/a	Involved in the study	
☒	☐	Antibodies
☒	☐	Eukaryotic cell lines
☒	☐	Palaeontology and archaeology
☐	☒	Animals and other organisms
☒	☐	Human research participants
☒	☐	Clinical data
☒	☐	Dual use research of concern

Methods

n/a	Involved in the study	
☒	☐	ChIP-seq
☒	☐	Flow cytometry
☒	☐	MRI-based neuroimaging

Animals and other organisms

Policy information about studies involving animals; ARRIVE guidelines recommended for reporting animal research

Laboratory animals	None
Wild animals	Fouling marine invertebrate species (Fenestrulina rugula, Celleporella antarctica, Celleporella dictyota, Micropora notialis, Chaperiopsis protecta, Tubulipora sp, Arachnopusia inchoata, Ellisina antarctica, Aimulosia antarctica, Lichenopora sp, Idmidronea sp, Beania errecta, Camptoplites bicornis, Filaguria spatulata, Smittina rogickae, Notoplites sp, Lageneschara lyrulata, Beania costata, Romanchella perrieri, Protolaeospira stalagmia, Paralaeospira levinseni, Corella eumyota, Cnemidocarpa verrucosa, Molgula sp, Pyura sp, Ascidia sp) which fouled plastic panels were collected and killed by exposure to air.
Field-collected samples	Not applicable
Ethics oversight	No ethical approval required

Note that full information on the approval of the study protocol must also be provided in the manuscript.
